# 

**Published:** 1972-04

**Authors:** 


					Bristol Medico-Chirurgical Journal Vol. 87 (ii) No. 322
Contents
Some Crises in Adolescence
W. Lumsden Walker
15
The Psychology of the Naturalist
Philip Radford
19
Obituary
21
Bristol Medico-Chirurgical Society
22
Printed by F. Bailey & Son Ltd., Dursley, Glos.

				

